# Early Recognition of Infantile Systemic Hyalinosis in a Palestinian Infant: A Case Report

**DOI:** 10.1002/ccr3.71126

**Published:** 2025-10-03

**Authors:** Lilyan Jarrar, Sara Mutan, Lana Malhis, Ahmad Mashni, Afnan Ajlone, Mahdi Zaid, Sara Abuaisha

**Affiliations:** ^1^ Jenin Governmental Hospital Jenin Palestine; ^2^ Faculty of Medicine and Health Sciences An‐Najah National University Nablus Palestine; ^3^ Al‐Watani Hospital Jenin Palestine

**Keywords:** Arabs, consanguinity, genetic testing, hyaline fibromatosis syndrome

## Abstract

Infantile systemic hyalinosis should be suspected in infants with contractures, skin changes, and diarrhea in consanguineous families. Early recognition allows timely genetic testing, supportive care, and counseling, improving family outcomes despite poor prognosis.

**Abbreviations**
ALT: alanine aminotransferase
AMC: arthrogryposis multiplex congenita
ANTXR2: anthrax toxin receptor 2
AST: aspartate aminotransferase
INR: international normalized Ratio
ISH: infantile systemic hyalinosis
IVIG: intravenous immunoglobulin
JHF: juvenile hyaline fibromatosis
NICU: neonatal intensive care unit
NOMID: neonatal‐onset multisystem inflammatory disease
PICU: pediatric intensive care unit
PT: prothrombin time
WBCs: white blood cells

## 1 Introduction

Infantile systemic hyalinosis (ISH) is a rare, autosomal recessive connective tissue disorder resulting from mutations in the ANTXR2 gene on chromosome 4q21. This gene encodes capillary morphogenesis protein‐2, which plays a key role in extracellular matrix stability. Its dysfunction leads to abnormal hyaline material deposition in the skin, joints, and internal organs. Clinically, ISH typically presents within the first year of life with gingival hypertrophy, painful joint contractures, recurrent diarrhea, and thickened, hyperpigmented skin over pressure points. ISH represents the severe end of the hyaline fibromatosis spectrum, sharing histologic features with juvenile hyaline fibromatosis but with earlier onset and more widespread systemic involvement [1, 2].

Although there is currently no curative treatment, early recognition is crucial for supportive management and timely genetic counseling [3]. This report describes an 8‐month‐old Palestinian male presenting with progressive joint contractures, gingival hypertrophy, recurrent diarrhea, and hyperpigmented skin lesions. Born to consanguineous parents and with a family history of early infant deaths, early genetic testing was pursued and confirmed a pathogenic ANTXR2 mutation, establishing the diagnosis of ISH. This case underscores the importance of clinical suspicion in facilitating early diagnosis of this rare condition.

## 2 Case Presentation/Examination

We present the case of an 8‐month‐old Palestinian male infant was referred to our facility with a 5 days history of persistent diarrhea, hypoactivity, and multiple joint contractures. His prenatal history was uneventful. A detailed fetal ultrasound was conducted and showed no abnormalities. There was no history of maternal teratogenic exposure. He was born at term via cesarean section with a birth weight of 2800 g, and both echocardiography and transfontanelle ultrasound were normal for his age. Apgar scores were 9 at both 1 and 5 min. Head circumference at 5th percentile, both weight and height at 3rd percentile.

The patient was admitted to the neonatal intensive care unit (NICU) shortly after birth due to generalized stiffness. Neonatal examination revealed a weak Moro reflex, intense crying upon joint movement, and fixed flexed knees. Orthopedic consultation raised the suspicion of Arthrogryposis Multiplex Congenita (AMC), and physiotherapy was initiated. There is no significant past medical history prior to the current illness.

**FIGURE 1:**
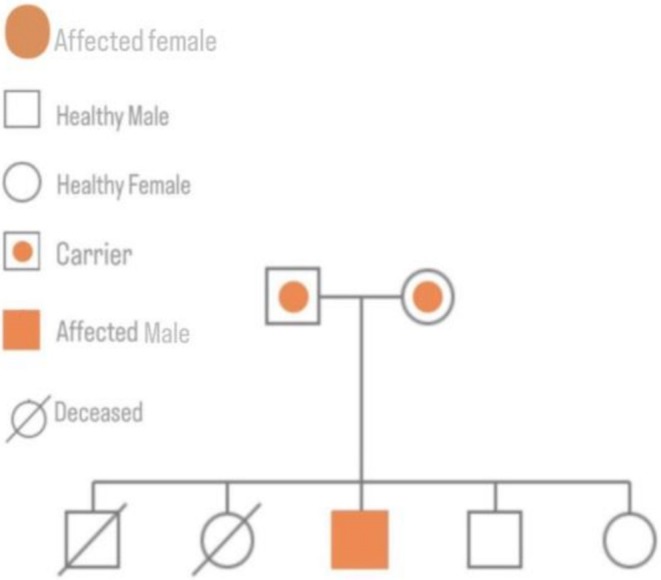
Family pedigree demonstrating autosomal recessive inheritance with consanguinity. Affected individuals are shown in orange, carriers are half‐shaded, and deceased individuals are marked with a diagonal line.

The family history is notable for consanguinity between the parents. Two previous siblings died at the ages of 7 and 6 months, both exhibiting joint contractures and recurrent infections. However, no other known genetic disorders have been reported in the extended family. Figure 1 depicts the family pedigree, highlighting a pattern suggestive of a possible inherited condition.

During the most recent hospitalization, the infant presented with watery diarrhea (six times daily for 5 days), fever (38.6°C axillary), signs of dehydration, and hypoactivity. Due to severe dehydration and collapsed peripheral veins, intravenous access was challenging. Given the limited resources, a femoral central line was inserted in the Pediatric Intensive Care Unit (PICU) after failed attempts at peripheral and intraosseous access.

**FIGURE 2:**
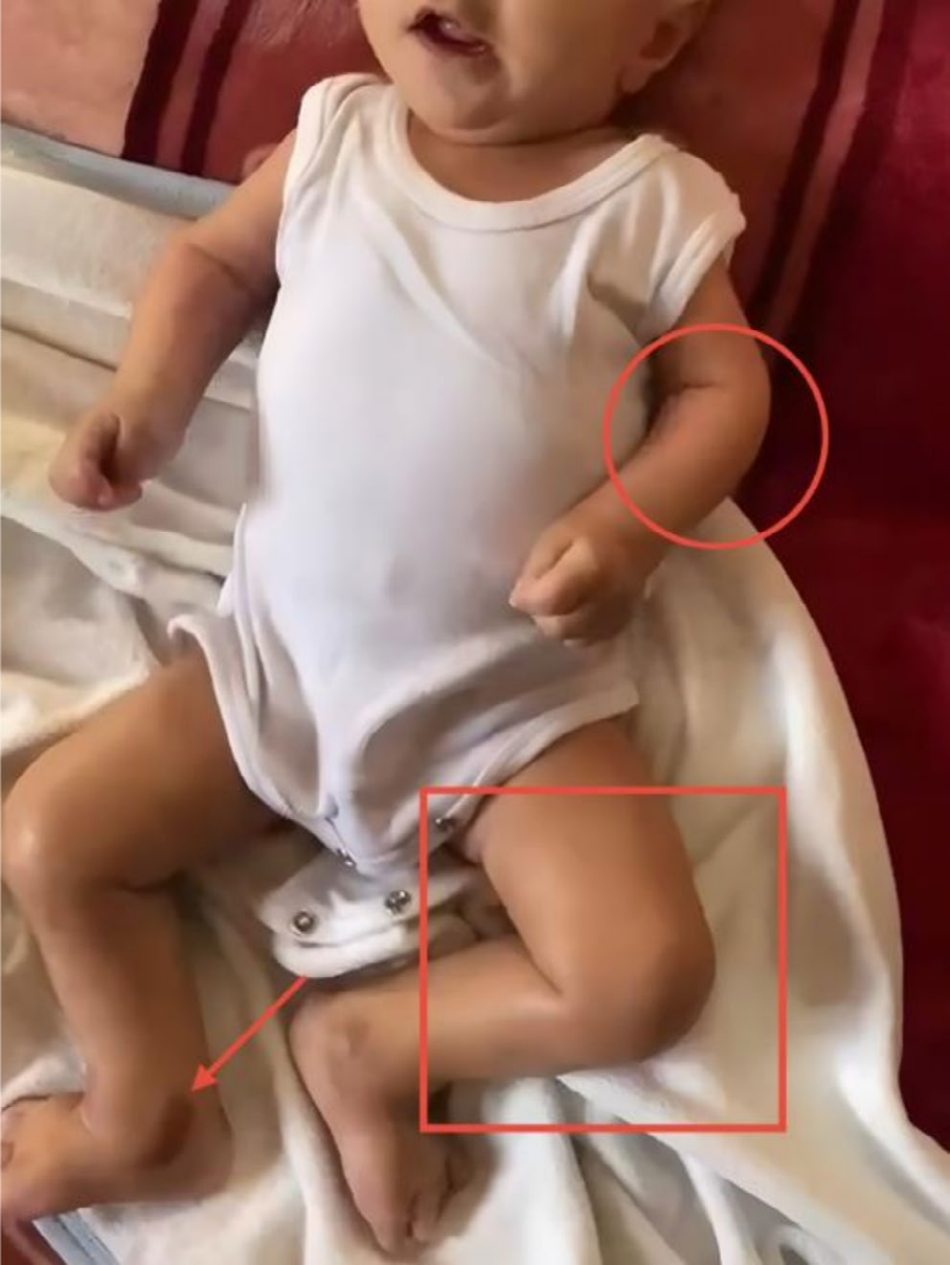
Clinical features of the patient. The red circle highlights elbow joint contracture, the red square indicates knee joint contracture, and the red arrow points to areas of skin hyperpigmentation.

On examination, the infant appeared ill and in pain with multiple hyperpigmented skin lesions (especially over his ankles and knees) and gingival hypertrophy without any skin nodules. Gastrointestinal examination revealed a soft abdomen with hyperactive bowel sounds. Musculoskeletal examination showed significant joint tenderness and contractures in the elbows (about 90 degrees), knees (about 90 degrees), and ankles. Neurological assessment was limited due to pain on movement (Figure 2). The clinical features are further illustrated in Video 1.

**VIDEO 1:** clinical features of an infant diagnosed with Infantile Systemic Hyalinosis. The infant is shown lying supine, maintaining a stiff posture with minimal spontaneous movements, consistent with joint contractures. There is visible skin discoloration on the medial aspects of both ankles. The elbows and knees appear markedly contracted. Video content can be viewed at https://onlinelibrary.wiley.com/doi/10.1002/ccr3.71126.

## 3 Methods

### 3.1 Differential Diagnosis

The differential diagnosis for Infantile Systemic Hyalinosis (ISH) includes several rare genetic and metabolic disorders. This includes mucopolysaccharidoses; in this patient, the absence of facial coarse features, organomegaly, and corneal clouding made this diagnosis less likely.

Winchester syndrome was a differential diagnosis too, because ISH and Winchester syndrome both share the thickened, hyperpigmented skin, However Winchester lacks papules and nodules which are characteristic for ISH; also, the absence of Winchester coarse facial features made it less likely. Eventually, the genetic testing for our patient revealed a mutation in the ANTXR2 gene which is specific for ISH.

### 3.2 Investigations

His family history raised clinical suspicion for ISH, prompting whole exome sequencing. Genomic amplification of exon 12 of ANTXR2 gene and direct sequencing to detect the presence of c.1041 + 5 G > C, IVS12 + 5 G > C mutation in intron 12 were performed on the DNA extracted from peripheral blood sample for the above mentioned patient, confirming the diagnosis. Laboratory tests revealed hyponatremia, hypoalbuminemia, elevated INR, and leukocytosis. Stool cultures were negative, Table 1. Abdominal ultrasound was unremarkable.

**TABLE 1:** Overview of the patient's laboratory workup.

	Before management	After management	Normal range
Total protein	5	7	6.6–8.7 g/dL
Albumin level	1.48 g/dL	3.84 g/dL	3.5–5.0 g/dL
Chloride	99	102	98–110 mmol/L
Potassium	3.6	4.9	3.5–5.3 mmol/L
Sodium	119	135	135–145 mmol/L
AST	28	30	0–50 U/L
ALT	22.6	22	0–41 U/L
INR	2.5	1.3	
PT	17.4	—	11–15 s
WBCs	29	10.5	4.6–11 K/uL

### 3.3 Management

Management included intravenous normal saline (initial bolus of 100 mL followed by D5 0.9% NaCl at 700 mL/24 h), potassium chloride (2 mEq/100 mL), intravenous albumin (5 g over 4 h), furosemide (6 mg concomitantly with albumin), and vitamin K. Pediatric gastroenterology consultation suggested protein‐losing enteropathy and advised albumin infusion in cases of fluid overload, zinc acetate 5 mg daily for 2 months, and initiation of Neocate formula.

A pediatric gastroenterologist was consulted and suspected protein‐losing enteropathy. It was recommended to continue albumin infusions in the presence of fluid overload regardless of serum albumin levels. Zinc acetate (5 mg daily for 2 months) was initiated, and the patient was started on Neocate formula. Given the increased susceptibility to infections in ISH patients and the ongoing immunoglobulin loss through the gut, IVIG was used as a supportive measure Intravenous immunoglobulin (IVIG) was scheduled every 3–4 weeks.

## 4 Discussion

Infantile Systemic Hyalinosis (ISH) is a rare, autosomal‐recessive connective tissue disorder characterized by abnormal deposition of hyaline material in multiple organs, leading to severe multi‐systemic dysfunction. Its prevalence is estimated to be less than 1 in 1,000,000 individuals worldwide [2]. As an autosomal recessive disorder, biallelic mutations in the Anthrax Toxin Receptor 2 (ANTXR2) gene, also known as Capillary Morphogenesis Gene 2 (CMG2), located on chromosome 4q21, are necessary for the development of ISH [1]. This gene plays a critical role in the interaction of proteins and other molecules to strengthen and support connective tissues such as bone, tendons, and ligaments. Mutations affecting the extracellular protein binding domain of capillary morphogenesis protein‐2 disrupt this function, leading to widespread hyaline accumulation within connective tissues [4].

Due to the autosomal recessive inheritance pattern, ISH has been reported across various populations globally but appears disproportionately prevalent among Arabs, Japanese, and Indians, likely due to higher rates of consanguinity which strongly supports the role of consanguinity in increasing the risk of homozygous pathogenic ANTXR2 variants [1]. This aligns with our case of an 8‐month‐old Palestinian male, born to consanguineous parents, with a family history of early infant deaths.

Clinically, ISH typically presents at birth or within the first 6 months of life. The hallmark features include painful joint contractures resulting in restricted movement, thickened and hyperpigmented skin over bony prominences, gingival hypertrophy, recurrent diarrhea, failure to thrive, and susceptibility to recurrent infections [1, 5]. Additional dermatological manifestations may include loss of skin elasticity, small pearly papules (especially on the face, scalp, and neck), and perianal fleshy nodules. In our patient, the progressive joint contractures, recurrent diarrhea, gingival hypertrophy, and hyperpigmented skin at pressure points closely mirror these classical clinical features, however, our case is notable for the early onset and rapid clinical decline, consistent with the most severe end of the spectrum. Unlike milder cases that presented with isolated cutaneous nodules or late joint involvement, our patient exhibited widespread systemic symptoms before 8 months of age, underscoring the aggressive disease course. Furthermore, the presence of multiple sibling deaths at similar ages within the same family highlights a particularly severe familial phenotype not commonly detailed in existing literature.

The differential diagnosis for Infantile Systemic Hyalinosis (ISH) includes mucopolysaccharidoses (types I and II), Winchester syndrome, neonatal‐onset multisystem inflammatory disease (NOMID), and congenital muscular dystrophies. Mucopolysaccharidoses are distinguished by coarse facial features, organomegaly, and corneal clouding, which were absent in our patient. Winchester syndrome was considered due to skin thickening but was less likely given the presence of nodular lesions and absence of skeletal dysplasia. NOMID and congenital muscular dystrophies were excluded based on the lack of systemic inflammation, CNS involvement, and muscle weakness. The diagnosis of ISH was confirmed through genetic testing, identifying a pathogenic ANTXR2 mutation [6].

Although ISH can be diagnosed based on clinical suspicion, confirmation via genetic testing or histopathological examination is crucial. Histologic evaluation typically reveals deposits of amorphous, fibrillar hyaline material in the mucosa and cutaneous tissues [7]. In our case, early genetic testing confirmed a pathogenic mutation in the ANTXR2 gene, solidifying the diagnosis.

ISH is classified within the hyaline fibromatosis syndrome spectrum, with ISH representing the severe end and Juvenile Hyaline Fibromatosis (JHF) the milder form. Despite their shared histological features, ISH typically manifests within the first few months of life and includes visceral organ involvement and life‐threatening complications, unlike JHF, which presents later with more localized disease and longer survival. Our case falls distinctly at the severe ISH end of the spectrum due to the early onset, presence of diarrhea, failure to thrive, and early mortality among siblings [5].

Currently, there is no definitive cure for ISH. Management is supportive and palliative, requiring a multidisciplinary team approach. Early physiotherapy can help mitigate joint contractures, surgical excision of painful nodules may be considered, and aggressive nutritional support is vital to address failure to thrive. Genetic counseling is imperative for affected families to understand recurrence risks and future reproductive options [3]. Early diagnosis, as highlighted in our case, can significantly impact the quality of supportive care provided.

Infantile Systemic Hyalinosis (ISH) carries a poor prognosis, primarily due to progressive multi‐organ dysfunction, recurrent infections, and intractable diarrhea leading to malnutrition and failure to thrive. Most affected individuals succumb within the first 2 years of life, with common causes of mortality including severe diarrhea, malabsorption, recurrent infections, and organ failure [8]. In a study of 19 patients, 16 died at a mean age of 11 months, and only three survived beyond 20 months [9]. Long‐term survival is exceedingly rare, although a few cases of survival into adulthood have been reported, often with significant physical disabilities due to joint contractures and limited mobility [10]. This grim prognosis was reflected in our patient's family history, where two siblings died at 6 and 7 months of age, respectively. The main limitation of this case report is the inability to provide long‐term follow‐up due to the rapid deterioration of the patient's condition. Although genetic testing confirmed the diagnosis, limited access to longitudinal clinical outcomes restricts a deeper understanding of disease progression and response to supportive care strategies.

This case emphasizes the critical importance of maintaining a high index of suspicion for ISH, especially in populations with prevalent consanguinity. Reporting such cases enriches the sparse clinical literature and underscores the necessity for heightened awareness among healthcare providers for timely diagnosis, intervention, and counseling.

## 5 Results (Outcome and Follow‐Up)

During hospitalization, the patient initially showed partial improvement after albumin infusions, IVIG therapy, and initiation of Neocate formula, with stabilization of electrolyte disturbances and modest gains in serum albumin. Despite these measures, his overall condition remained fragile, with persistent malnutrition, recurrent infections, and progressive clinical decline. Given the family history of two siblings who had died at similar ages with comparable symptoms, the prognosis in this case was considered extremely poor. Unfortunately, due to the rapid deterioration of the disease course, long‐term follow‐up could not be obtained.

## 6 Conclusion

Infantile Systemic Hyalinosis remains a devastating and life‐limiting disorder, particularly in populations with high rates of consanguinity. Early clinical suspicion, reinforced by genetic testing, plays a crucial role in diagnosing this rare condition. Although no curative treatment currently exists, early recognition enables more effective supportive care, helps manage complications, and allows for timely genetic counseling to affected families. Through reporting such cases, we aim to enhance awareness among healthcare professionals, which is essential for prompt diagnosis, optimal care, and future research into potential therapies.

## Author Contributions

**Lilyan Jarrar:** conceptualization, data curation, writing – original draft, writing – review and editing. **Sara Mutan:** writing – review and editing. **Lana Malhis:** writing – review and editing. **Ahmad Mashni:** writing – review and editing. **Afnan Ajlone:** writing – review and editing. **Mahdi Zaid:** conceptualization, resources. **Sara Abuaisha:** writing – review and editing.

## Ethics Statement

This study is a single‐patient case report and did not require institutional ethical approval.

## Consent

Written (journal form) and verbal informed consent for publication of clinical details and/or images were obtained from the patient's legal guardian in both Arabic and English. A copy of the consent form is available for review by the journal's editorial office upon request. Informed consent was obtained from the patient's legal guardian (her father), ensuring anonymity.

## Conflicts of Interest

The authors declare no conflicts of interest.

## Data Availability

All data generated or analyzed during this study are included in this published article. Additional data, such as laboratory values and the patient consent form, are available from the corresponding author upon reasonable request.
